# Supplementary material for: A RuBisCO-mediated carbon metabolic pathway in methanogenic archaea
Source: Nat Commun. 2017 Jan 13;8:14007. doi: 10.1038/ncomms14007 (PMC5241800; doi:10.1038/ncomms14007)
Supplement: Supplementary Information — Supplementary Figures, Supplementary Tables and Supplementary References [file ncomms14007-s1.pdf]

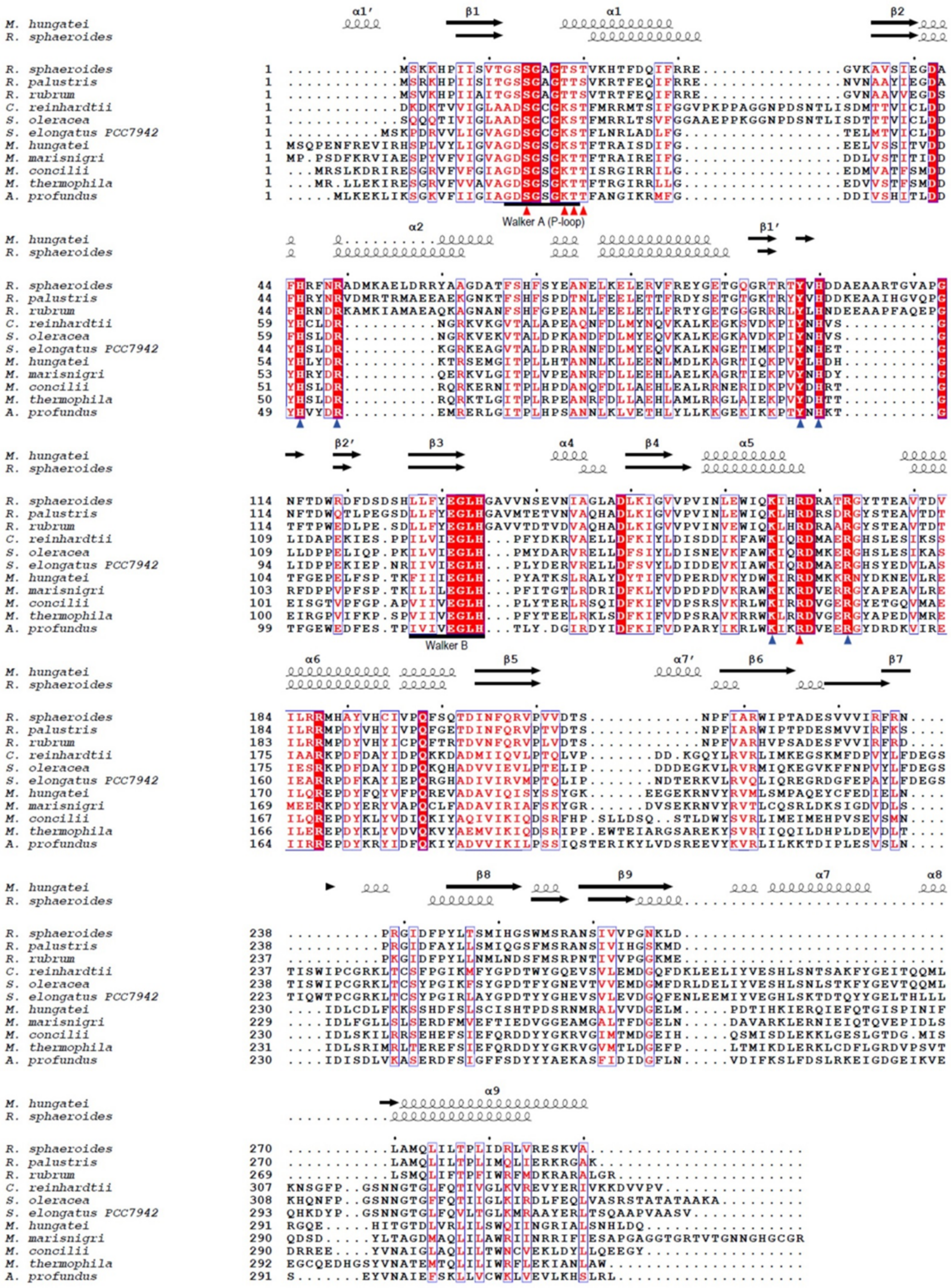
M. hungatei
R. sphaeroides
R. palustris
R. rubrum
C. reinhardtii
S. oleracea
S. elongatus PCC7942
M. marisnigri
M. concilii
M. thermophila
A. profundus
α1′
β1
α1
β2
Walker A (P-loop)
α2
β1′
β2′
β3
α4
β4
α5
Walker B
α6
β5
α7′
β6
β7
β8
β9
α7
α8
α9

Supplementary Figure 1: Sequence alignment of archaeal and photosynthetic PRKs. Sequences are those of five archaeal PRKs from *M. hungatei* (ABD40546), *M. marisnigri* (ABN57860), *M. concilii* (AEB68483), *M. thermophila* (ABK15221), and *A. profundus* (ADB58593); three photosynthetic bacterial PRKs from *R. sphaeroides* (AAA26113), *R. palustris* (ADU46366), and *R. rubrum* (ABC23204); and three plant-type PRKs from *S. oleracea* (CAA30499), *C. reinhardtii* (AAF36402), and *S. elongatus* PCC 7942 (ABB57007). Secondary-structure assignments above sequences are derived from structures of PRKs in *M. hungatei* (PDB ID 5B3F and *R. sphaeroides* (PDB ID 1A7J). Greek characters denote α-helices and β-strands. Residue numbers are shown on the right side of sequences. Red boxes highlight fully conserved residues. Red amino acid residues are moderately conserved. Black bars show Walker A (P-loop) and B motifs. Residues involved in binding of Ru5P and ATP are marked by red and blue triangles, respectively, based on mutagenesis analyses of RsPRK[1-5].

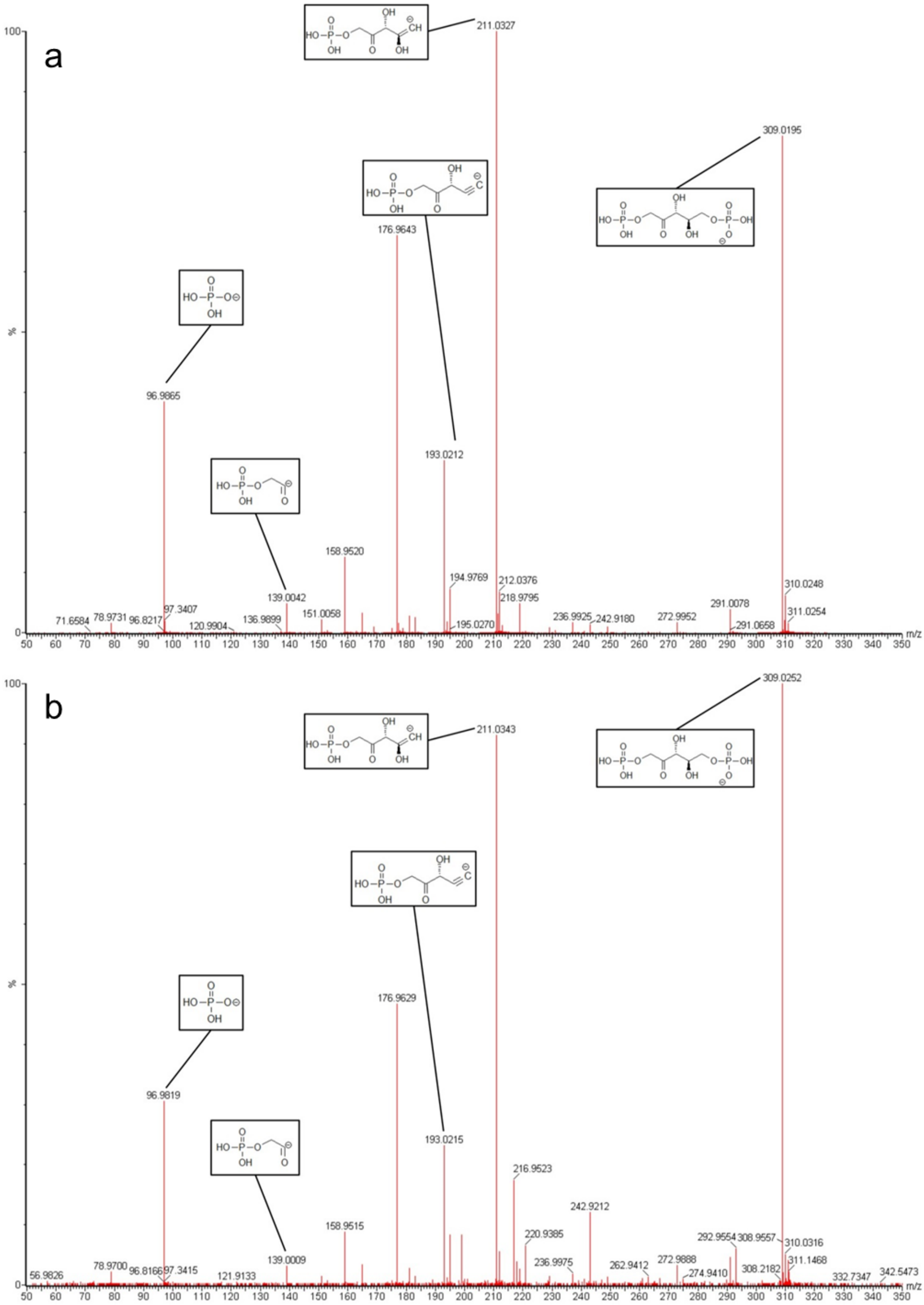

Supplementary Figure 2: ESI-MS/MS analysis of the reaction product of MhPRK.

Spectra of (**a**) authentic RuBP and (**b**) the MhPRK reaction product.

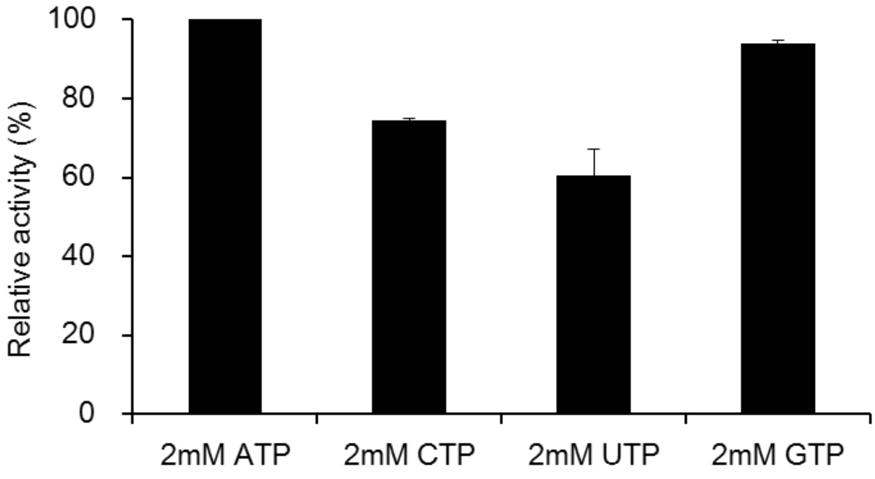

Supplementary Figure 3: Nucleoside triphosphate (NTP) specificity of MhPRK.

In the assay for NTP specificity of *M. hungatei* PRK, ATP was replaced with the following substrates: 2 mM CTP, 2 mM UTP, or 2 mM GTP[6,7]. The vertical line represents the ratio of the activity of the NTPs to that of ATP. Data are means ± SD of three replicates.

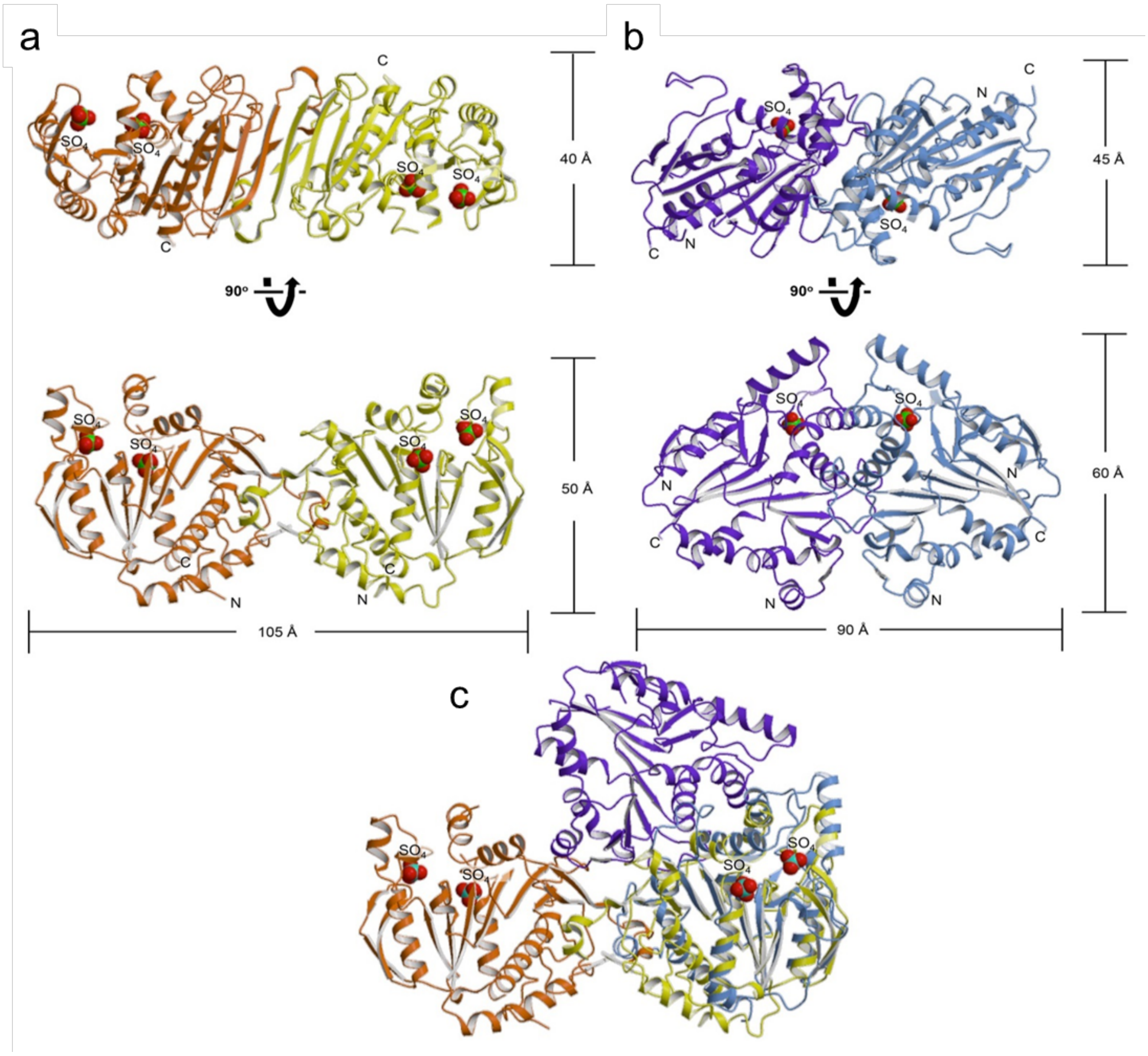

Supplementary Figure 4: Structural comparison of MhPRK and RsPRK dimers.

Ribbon diagrams of (**a**) the MhPRK dimer and (**b**) the RsPRK dimer in two orientations related by a 90° rotation around a horizontal axis. (**c**) One chain of the photosynthetic RsPRK dimer superimposed on that of the MhPRK dimer (RsPRK assembles as a tetramer of dimers). Chains (**a)** and (**b)** are shown in orange and yellow in MhPRK, and purple and blue in RsPRK, respectively. Sulphate ions are bound to the phosphate-binding pockets in the active sites of MhPRK.

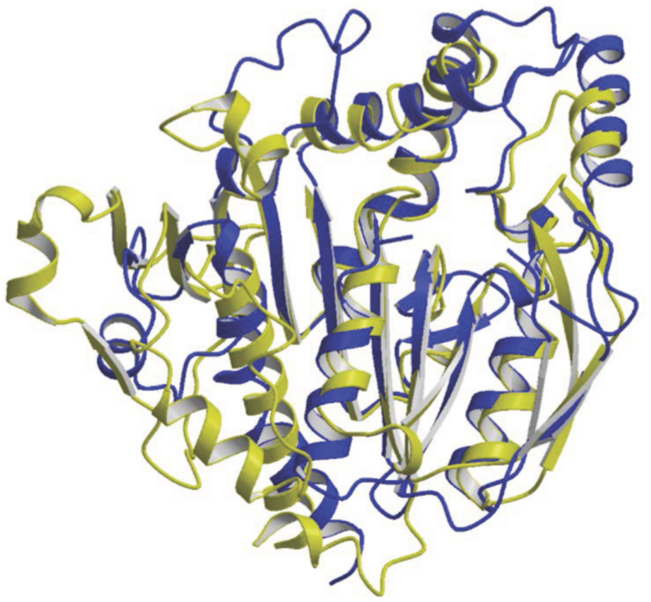

Supplementary Figure 5: Structural superposition of MhPRK and RsPRK.

MhPRK (PDB ID 5B3F) and RsPRK (PDB ID 1A7J) are shown in yellow and blue, respectively. The N-terminal domain (1–198) of MhPRK resembles that of RsPRK, with an overall r.m.s. ds of 4.1 Å over 203 equivalent Cα positions for RsPRK; in comparison, the C-terminal domain (199–319 for MhPRK) is relatively diverse (Supplementary Fig. 1).

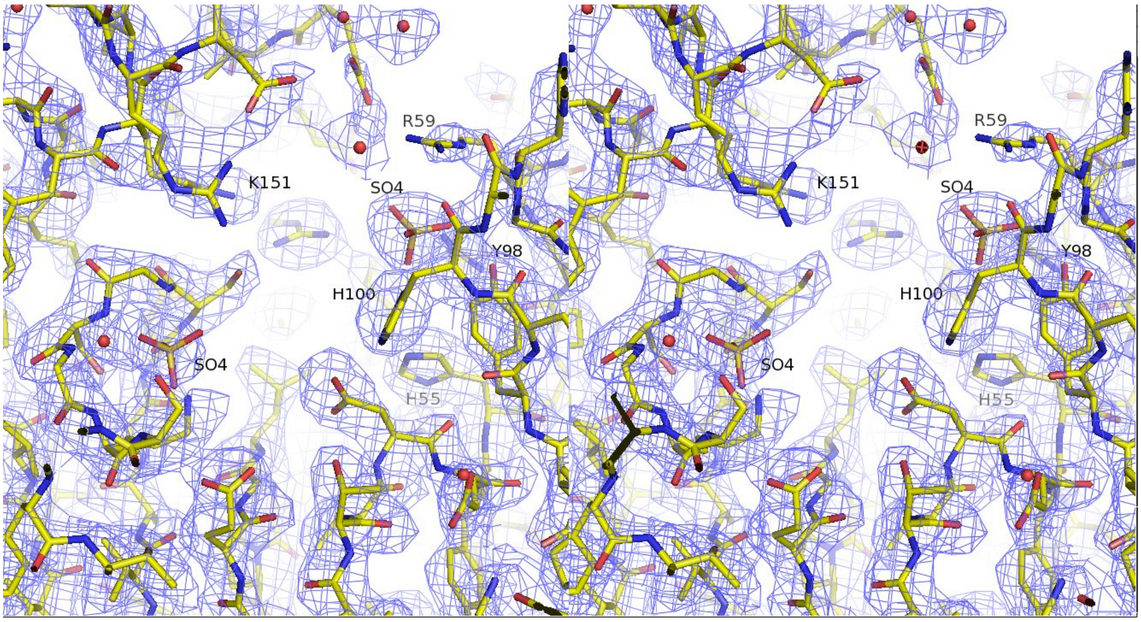

Supplementary Figure 6: Stereo view of the electron density map of MhPRK.

Stereo views (cross-eyed) of the refined 2Fo − Fc electron density map (contoured at 1.0 σ) at the active site of MhPRK.

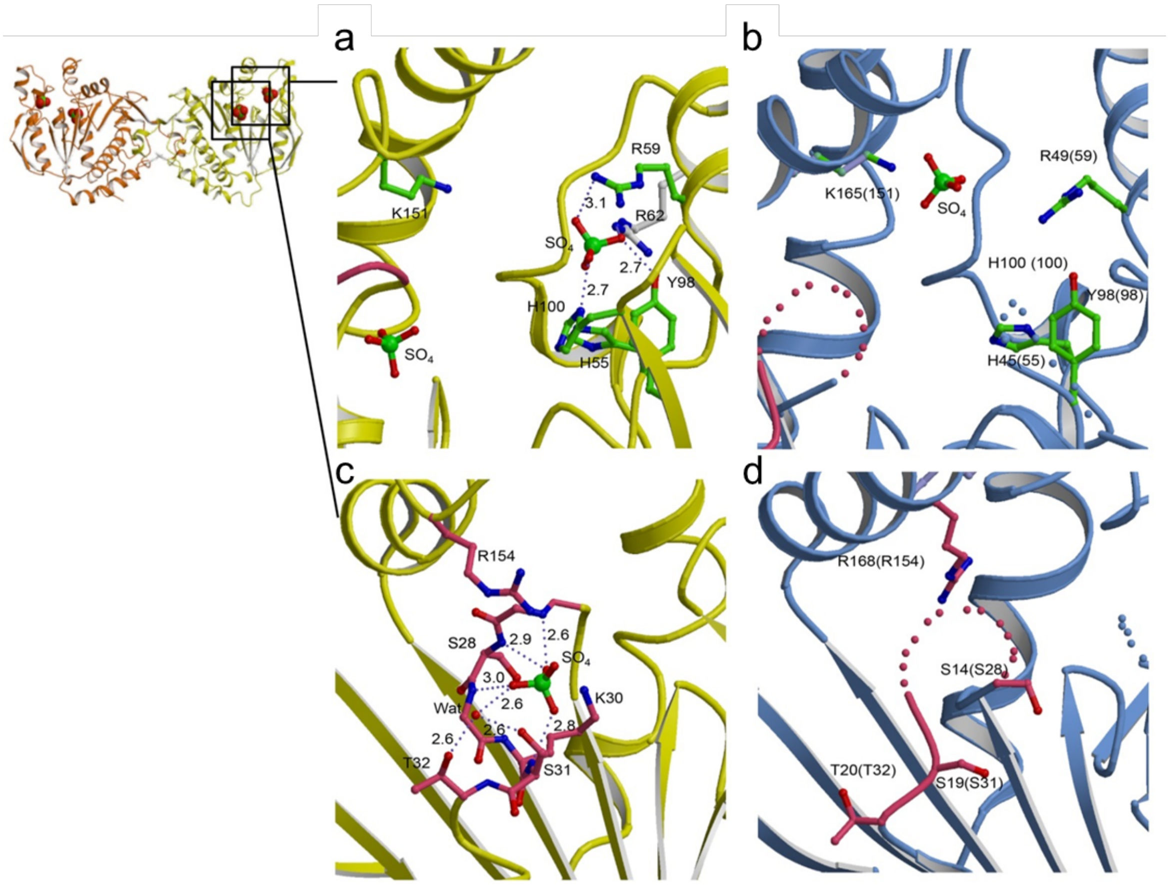

Supplementary Figure 7: Comparison of active sites between MhPRK and RsPRK.

Active sites around Ru5P-binding and ATP-binding sites in MhPRK (**a** and **c**) and in RsPRK (**b** and **d**). Side chains of residues, sulphate ions, water molecules, and disordered regions of RsPRK are depicted in the same manner as in Figure 1. For MhPRK, interactions of active site sulphate ions are shown as dotted lines with distance in Å. For RsPRK, numbers in parentheses represent corresponding amino acid residue in MhPRK.

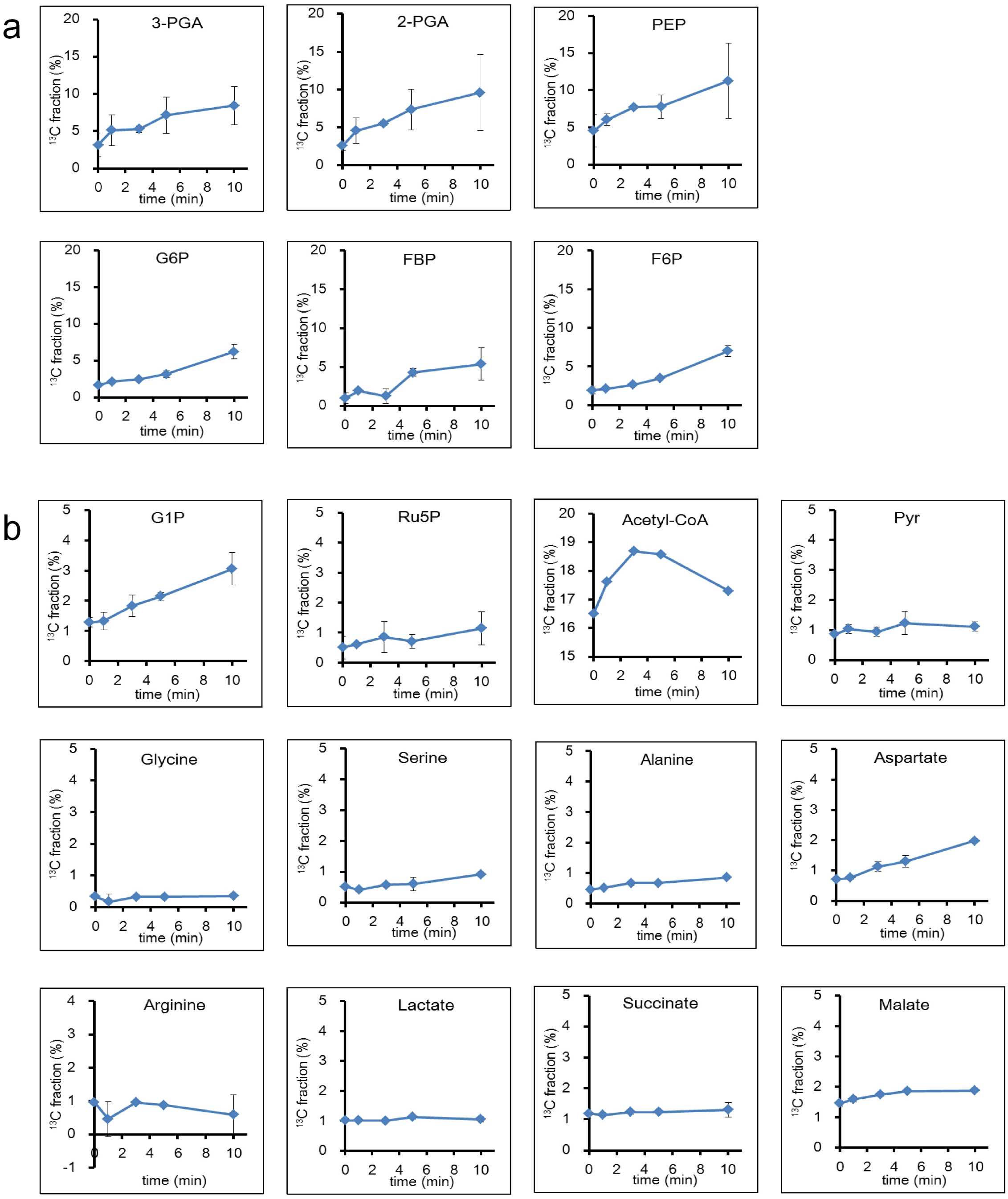

Supplementary Figure 8: Time-course analysis of the metabolite ^{13}C fraction of *M. hungatei* cells.

Metabolites with high and low ^{13}C labelling rate are shown in a and b, respectively. The vertical line represents the ratio of ^{13}C to total carbon in each metabolite. Data are means ± SD of two replicates. 3-PGA, 3-phosphoglycerate; 2-PGA, 2-phosphoglycerate; PEP, phosphoenolpyruvate; G6P, glucose-6-phosphate; FBP, fructose-1,6-bisphosphate; F6P, fructose-6-phosphate; G1P, glucose-1-phosphate; Ru5P, ribulose-5-phosphate; Pyr, pyruvate.

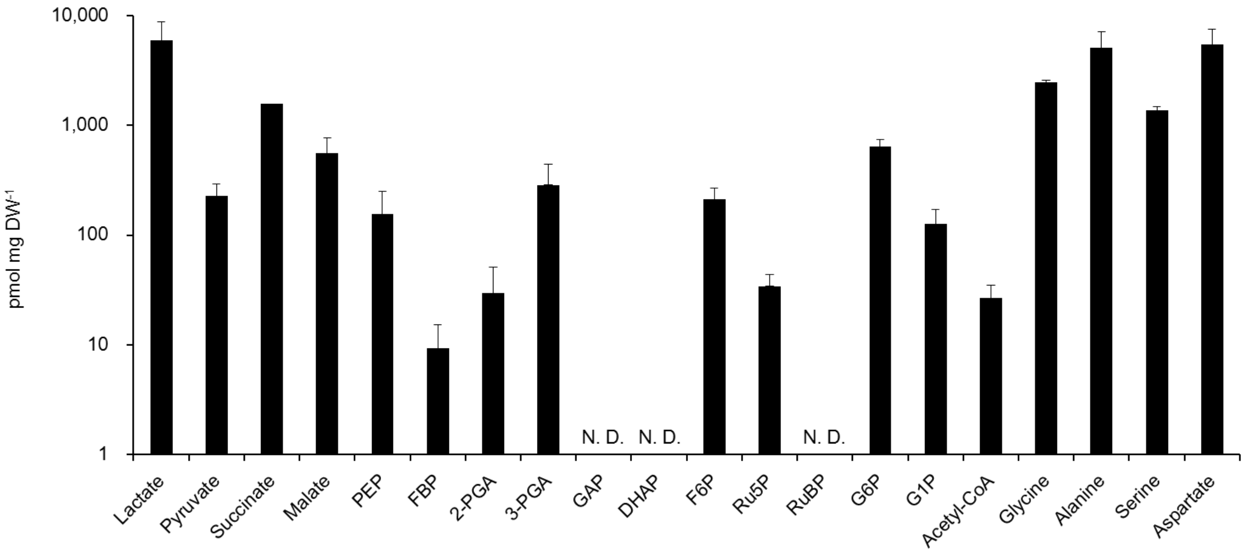

Supplementary Figure 9: Pool sizes of metabolites of *M. hungatei* cells.

The original pool sizes of unlabelled metabolites of *M. hungatei* cells were detected by CE/MS analysis. The vertical line represents the amount of each metabolite (picomole) per dry weight cell (mg). Data are means ± SD of two replicates. N.D., not detected. PEP, phosphoenolpyruvate; FBP, fructose-1,6-bisphosphate; 2-PGA, 2-phosphoglycerate; 3-PGA, 3-phosphoglycerate; GAP, glyceraldehyde-3-phosphate; DHAP, dihydroxyacetone phosphate; F6P, fructose-6-phosphate; Ru5P, ribulose-5-phosphate; RuBP, ribulose-1,5-bisphosphate; G6P, glucose-6-phosphate; G1P, glucose-1-phosphate.

Supplementary Table 1: Distribution of enzymes in the RHP pathway, the pentose bisphosphate pathway and the reductive acetyl-CoA pathway in members of the Archaea.

	RHP pathway						Reductive acetyl-CoA pathway						
				Pentose bisphosphate pathway									
Species	PRK	HPS	PHI	RuBisCO	AMP phosphorylase	RiBP isomerase	Fae	FMD	FTR	MCH	MTD	MER	CODH/ ACS
Aciduliprofundum boonei	•	•	•	•	•	•							
Archaeoglobus fulgidus		•	•	•	•	•	• *	•	•	•	•	•	•
Archaeoglobus profundus	•	•	•	•	•	•	• *		•	•	•	•	
Archaeoglobus veneficus	•	•	•	•	•	•	• *	•	•	•	•	•	•
Ferroglobus placidus	•	•	•	•	•	•	•	•	•	•	•	•	•
Methanocaldococcus janaschii		•	•	•	•	•	• *	•	•	•	•	•	•
Methanococcoides burtonii		•	•	•	•	•	•	•	•	•	•	•	•
Methanococcus maripaludis		•	•		•	•		•	•	•	•	•	•
Methanoculleus marisnigri	•	•	•	•	•	•	•	•	•	•	•	•	•
Methanofollis liminatans	•	•	•	•	•		• *	•	•	•	•	•	•
Methanolinea tarda	•	•	•	•	•	•	• *	•	•	•	•	•	•
Methanoplanus limicola	•	•	•	•	•	•	• *	•	•	•	•	•	
Methanoplanus petrolearius	•	•	•	•	•	•	• *	•	•	•	•	•	
Methanoregula boonei	•	•	•	•	•		• *	•	•	•	•	•	•
Methanosaeta concilii	•	•	•	•	•	•	•	•	•	•	•	•	•
Methanosaeta harundinacea	•	•	•	•	•	•	•	•	•	•	•	•	•
Methanosaeta thermophila	•	•	•	•	•	•	• *	•	•	•	•	•	•
Methanosarcina acetivorans		•	•	•	•	•	•	•	•	•	•	•	•
Methanosphaerula palustris	•	•	•	•	•		• *	•	•	•	•	•	•
Methanospirillum hungatei	•	•	•	•	•	•	• *	•	•	•	•	•	•
Natronomonas pharaonis				•	•	•				•		•	
Pyrococcus horikoshii		•	•	•	•	•							
Sulfolobus tokodaii		•	•			•							•
Thermococcus kodakarensis		•	•	•	•	•							

Filled circles indicate presence of a homolog gene for each enzyme. FMD, formylmethanofuran dehydrogenase; FTR, formylmethanofuran: tetrahydromethanopterin formyltransferase; MCH, methenyl-tetrahydromethanopterin cyclohydrolase; MTD, methylene-tetrahydromethanopterin dehydrogenase; MER, methylenetetrahydromethanopterin reductase; CODH/ACS, CO Dehydrogenase/Acetyl-CoA Synthase. * Fae fused HPS.

Supplementary Table 2: Specific activities of RuMP pathway enzymes in *M. hungatei*.

Enzyme	Specific activity (μmol^{-1} min^{-1} mg $protein^{-1}$)
HPS-MenG	0.21 ± 0.05
Fae-HPS	6.05 ± 1.64
PHI-a	N. D.
PHI-b	N. D.
PHI-a and -b	0.71 ± 0.17

Values are means ± SD (n = 3 replicates). N.D., not detected.

Supplementary Table 3: Primer sequences used in this study

Primer name	Primer sequence (5′–3′)
Mhprk-F	GATCCTGAACCTTCATATGAGTCAGCCTGAAAATTTCCG
Mhprk-R	GCGTATTATTTGAGGTACTCGAGTTATTGATCCAGATGATTGG
Mhrubisco-F	GCGAGGAAGCTCGAGATGACAGACGTTATTGCAAC
Mhrubisco-R	GGTATTTCCATGTAGGATCCTTATGCAATTCCCC
Mmprk-F	CAGCCATATGCTCGAGATGCCCCCATCCGAC
Mmprk-R	GTTAGCAGCCGGATCCTCACCTTCGGCCGCA
Mcprk-F	CAGCCATATGCTCGAGATGAGATCGCTCAAG
Mcprk-R	GTTAGCAGCCGGATCCCTAATAGCCCTCTTC
Mtprk-F	CAGCCATATGCTCGAGATGCGACTTCTCGAG
Mtprk-R	GTTAGCAGCCGGATCCTCACCAGGCGAGATT
Apprk-F	CAGCCATATGCTCGAGATGCTTAAAGAGAAG
Apprk-R	GTTAGCAGCCGGATCCCTACAATCTCAAACT
Mhfaehps-F	GCTCGGTACCCTCGAGATGTATCTGATAGGCGAA
Mhfaehps-R	GCTTGAATTCGGATCCTTAGAAATCAGTCATGATT
Mhhpsmeng-F	GCTCGGTACCCTCGAGATGAACCGGTCTGTACTC
Mhhpsmeng-R	GCTTGAATTCGGATCCTCAGGAATGTTTTTCCCAC
Mhphi-a-F	GCTCGGTACCCTCGAGATGCAACTGATGGCGTCA
Mhphi-a-R	GCTTGAATTCGGATCCTCACTCGATATTTGCATGC
Mhphi-b-F	GCTCGGTACCCTCGAGATGTCCATGATGATCTCC
Mhphi-b-R	GCTTGAATTCGGATCCTTATTGCATATTGGTATAC

F – forward, R - reverse

Supplementary Table 4: Data collection, phasing and refinement statistics for SAD (SeMet) structures.

	Native	SeMet
**Data collection**		
Space group	$P2_12_12_1$	$P3_121$
Cell dimensions		
a, *b*, *c* (Å)	78.41, 93.82, 99.94	99.49, 99.49, 171.80
a, *b*, *g* (º)	90.0, 90.0, 90.0	90.0, 90.0, 120.0
Wavelength	0.90	0.975
Resolution (Å)	50.0-2.5 (2.54-2.50) *	50.0-2.6 (2.64-2.60) *
R_{sym}	0.073 (0.274)	0.086 (0.370)
I/s*I*	13.5 (2.0)	17.2 (1.6)
Completeness (%)	92.2 (90.0)	88.7 (61.2)
Redundancy	2.9 (2.9)	5.1 (4.2)
**Refinement**		
Resolution (Å)	34.0-2.5	
No. reflections	23005	
$R_{work/}$ R_{free}	0.223 / 0.279	
No. atoms		
Protein	4979	
Ligand/ion	20	
Water	149	
B-factors		
Protein	45.0	
Ligand/ion	67.4	
Water	40.2	
R.m.s deviations		
Bond lengths (Å)	0.007	
Bond angles (º)	1.3	

Footnote: *Highest resolution shell is shown in parenthesis.

Supplementary References

1 Runquist, J. A. & Miziorko, H. M. Functional contribution of a conserved, mobile loop histidine of phosphoribulokinase. *Protein. Sci.* **15**, 837–842 (2006).

2 Sandbaken, M. G., Runquist, J. A., Barbieri, J. T. & Miziorko, H. M. Identification of the phosphoribulokinase sugar phosphate binding domain. *Biochemistry* **31**, 3715–3719 (1992).

3 Runquist, J. A., Harrison, D. H. & Miziorko, H. M. Functional evaluation of invariant arginines situated in the mobile lid domain of phosphoribulokinase. *Biochemistry* **37**, 1221–1226, doi:10.1021/bi972052f (1998).

4 Runquist, J. A., Harrison, D. H. & Miziorko, H. M. *Rhodobacter sphaeroides* phosphoribulokinase: identification of lysine-165 as a catalytic residue and evaluation of the contributions of invariant basic amino acids to ribulose 5-phosphate binding. *Biochemistry* **38**, 13999–14005 (1999).

5 Runquist, J. A., Rios, S. E., Vinarov, D. A. & Miziorko, H. M. Functional evaluation of serine/threonine residues in the P-loop of *Rhodobacter sphaeroides* phosphoribulokinase. *Biochemistry* **40**, 14530–14537 (2001).

6 Hurwitz, J., Weissbach, A., Horecker, B. L. & Smyrniotis, P. Z. Spinach phosphoribulokinase. *J. Biol. Chem.* **218**, 769–783 (1956).

7 Siebert, K., Schobert, P. & Bowien, B. Purification, some catalytic and molecular properties of phosphoribulokinase from *Alcaligenes eutrophus*. *Biochim. Biophys. Acta.* **658**, 35–44 (1981).
